# Deletion of the mitochondria-shaping protein *Opa1* during early thymocyte maturation impacts mature memory T cell metabolism

**DOI:** 10.1038/s41418-021-00747-6

**Published:** 2021-03-01

**Authors:** Mauro Corrado, Dijana Samardžić, Marta Giacomello, Nisha Rana, Erika L. Pearce, Luca Scorrano

**Affiliations:** 1 Veneto Institute of Molecular Medicine, Padua, Italy; 2 Max Planck Institute of Immunobiology and Epigenetics, Freiburg im Breisgau, Germany; 3 Department of Biology, University of Padua, Padua, Italy

**Keywords:** Immune cell death, T cells

## Abstract

Optic atrophy 1 (OPA1), a mitochondria-shaping protein controlling cristae biogenesis and respiration, is required for memory T cell function, but whether it affects intrathymic T cell development is unknown. Here we show that OPA1 is necessary for thymocyte maturation at the double negative (DN)3 stage when rearrangement of the T cell receptor β (*Tcrβ*) locus occurs. By profiling mitochondrial function at different stages of thymocyte maturation, we find that DN3 cells rely on oxidative phosphorylation. Consistently, *Opa1* deletion during early T cell development impairs respiration of DN3 cells and reduces their number. *Opa1*-deficient DN3 cells indeed display stronger TCR signaling and are more prone to cell death. The surviving *Opa1*^−/−^ thymocytes that reach the periphery as mature T cells display an effector memory phenotype even in the absence of antigenic stimulation but are unable to generate metabolically fit long-term memory T cells. Thus, mitochondrial defects early during T cell development affect mature T cell function.

## Introduction

The T cell repertoire develops from precursor cells migrating from the bone marrow into the thymus where a complex multistep maturation program generates functional CD4^+^ and CD8^+^ T cells able to discriminate self/non-self-antigen [1, 2]. In an early phase of thymocyte development, progenitors lacking expression of the major histocompatibility complex (MHC) co-receptors CD4 and CD8 are defined double negative (DN) cells and are classically divided into DN1–DN4 cells according to the expression of the surface markers CD44 and CD25 (DN1: CD44^high^CD25^low^; DN2: CD44^high^CD25^high^; DN3: CD44^low^CD25^high^; DN4: CD44^low^CD25^low^) [2, 3]. After preliminary lineage commitment, thymocytes initiate rearrangement of the T cell receptor (*Tcr*) *β* locus at the DN3 stage [4]. Only cells producing a functional T cell receptor β (TCRβ) chain are allowed to pass beyond DN3 stage, through a process called β-selection [2]. DN4 cells then undergo rapid proliferation and differentiate in CD4^+^ CD8^+^ double positive (DP) T cells, where TCRα chain rearrangement takes place [5, 6]. During the DP stage, only cells with a competent TCR that binds MHC (class I or II) with a low affinity are positively selected [2]. When TCR/MHC affinity is too high, activation-induced cell death (AICD) is triggered to avoid the generation of autoreactive T cells [2, 7]. The class identity of MHC molecules interacting with the TCR dictates the final commitment: T cells binding to MHC-II will become CD4^+^, whereas MHC-I-binding cells will mature into CD8^+^ T cells [2].

The first report describing the role of energy metabolism in thymocyte development dates back to 1978 [8]. Nevertheless, for many years the study of metabolism in thymocytes has been limited to the analysis of the effects of NOTCH and interleukin (IL)-7 signaling on cell survival and growth [6, 9, 10]. More recently, glucose and glutamine were shown to fuel protein O-GlcNAcylation during β-selection [11]. In addition, deletion of the mitochondrial pyruvate carrier 1, essential for pyruvate entry in the mitochondrial matrix and hence for fueling the tricarboxylic acid (TCA) cycle, impairs thymocyte development at different stages resulting in the generation of abnormally activated αβ T cells [12]. In line with that, the loss of RAPTOR-mediated mTORC1 activity also disturbs thymocyte metabolism and reciprocal lineage decision between αβ and γδ T cells [13, 14].

In response to intracellular and extracellular inputs, mitochondrial fusion and fission regulate respiration, apoptosis, and signaling to nucleus and other organelles [15–17]. Dynamin-related protein 1 (DRP1) is the main regulator of mitochondrial fission and in T cells promotes in vitro migration of lymphocytes, mitochondria localization at immunological synapse, and in vivo cMYC-dependent metabolic reprogramming and expansion upon activation [18–20]. Optic atrophy 1 (OPA1) is required for inner mitochondrial membrane (IMM) fusion, apoptosis, cristae morphology, organization of electron transport chain (ETC) supercomplexes and hence respiration [21–23]. In T cells, OPA1 is necessary to generate long-term T cell memory in secondary lymphoid organs [24]. Interestingly, both DRP1 and OPA1 coordinate AICD, pivotal for thymocyte negative selection and for effector cell contraction after infection [25–27].

Despite these observations, the role of mitochondrial function and of OPA1 during thymocyte development remains unknown. We found that among T cell precursors, DN3 cells are characterized by high oxidative phosphorylation (OXPHOS) capacity. Accordingly, OPA1 is required during thymocyte β-selection and *Opa1* deficiency alters TCR signaling in thymocytes, resulting in lymphopenia and impaired metabolism of mature T cells.

## Results

### High OXPHOS flags developing thymocytes during *Tcrβ* locus rearrangement

**Fig. 1:**
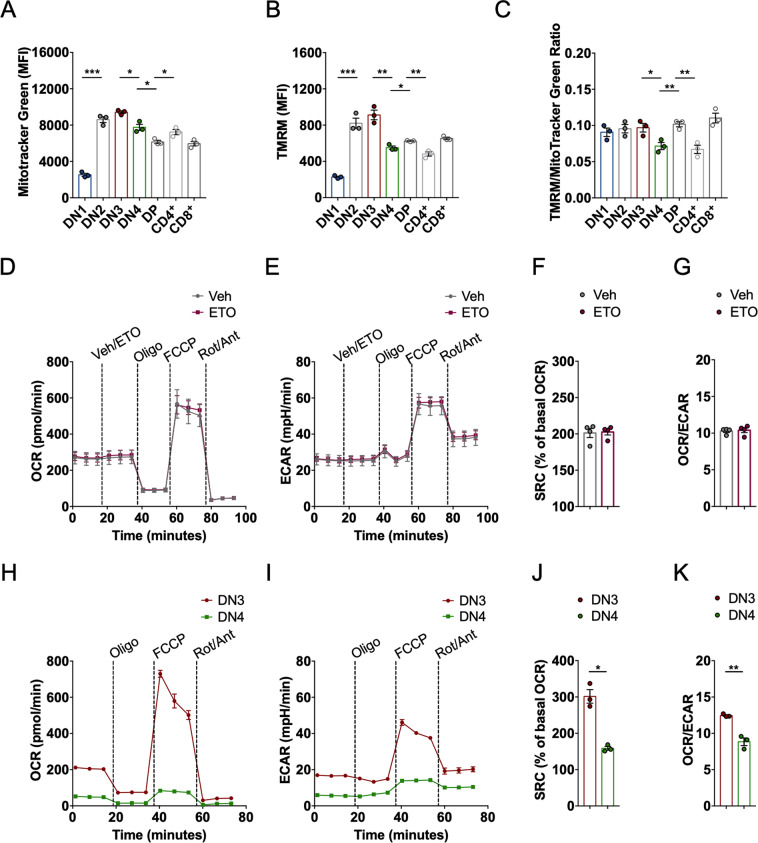
High OXPHOS flags developing thymocytes at β-selection stage. **A** Quantification of mitochondrial mass measured with MitoTracker Green in WT thymocytes analyzed by flow cytometry. Data are mean ± SEM (*N* = 3). **B** Quantification of mitochondrial membrane potential measured with TMRM in WT thymocytes analyzed by flow cytometry. Data are mean ± SEM (*N* = 3). **C** Mitochondrial membrane potential/mitochondrial mass ratio expressed as TMRM/Mitotracker green ratio in WT;Lck-Cre^+^ thymocytes analyzed by flow cytometry. Data are mean ± SEM (*N* = 3). **D** Oxygen consumption rate (OCR) of WT thymocytes at baseline and after exposure to vehicle/etomoxir, oligomycin (Oligo), FCCP, and rotenone/antimycin (Rot/Ant). Data are representative of three independent experiments (*N* = 4/group). **E** Extracellular acidification rate (ECAR) of WT thymocytes at baseline and after exposure to vehicle/etomoxir, oligomycin (Oligo), FCCP, and rotenone/antimycin (Rot/Ant). Data are representative of three independent experiments (*N* = 4/group). **F** SRC of WT thymocytes treated as in (**D**). Data are mean ± SEM (*N* = 4). **G** OCR/ECAR ratio of WT thymocytes treated as in (**D**). Data are mean ± SEM (*N* = 4). **H** WT DN3 and DN4 thymocytes were sorted and OCR was measured at baseline and after exposure to oligomycin (Oligo), FCCP, and rotenone/antimycin (Rot/Ant). Data are mean ± SEM (*N* = 3). **I** WT DN3 and DN4 thymocytes were sorted and ECAR was measured at baseline and after exposure to oligomycin (Oligo), FCCP, and rotenone/antimycin (Rot/Ant). Data are mean ± SEM (*N* = 3). **J** SRC of WT DN3 and DN4 thymocytes treated as in (**E**). Data are mean ± SEM (*N* = 3). **K** OCR/ECAR ratio of WT DN3 and DN4 thymocytes treated as in (**E**). Data are mean ± SEM (*N* = 3).

During thymocyte development, mitochondrial respiration appears to peak at the DN3 stage [12, 13], but it is unclear whether this is the epiphenomenon of mitochondrial biogenesis and increased mass, or due to specific mechanisms regulating mitochondrial fuel utilization/respiration. We therefore profiled mitochondrial membrane potential (Δψ) using the potentiometric dye tetramethyl rhodamine methyl ester (TMRM) and mitochondrial mass with the potential-insensitive staining MitoTracker Green in different subsets of thymocytes isolated from wild-type (WT) mice. Fluorescence of both dyes increased during the DN1 → DN3 transition, suggesting an increase in mitochondrial mass and mitochondrial respiratory capacity during β-selection, and then it dropped in DN4 thymocytes (Figs. 1A, B, S1A, and S1B). During the DN4 → DP transition, MitoTracker Green signal further decreased, whereas TMRM staining increased. In mature thymocytes, MitoTracker Green staining was higher in CD4^+^ than in CD8^+^ T cells; TMRM fluorescence conversely was lower in CD4^+^ T cells compared the CD8^+^ subset (Fig. 1A, B). The finding of a lower membrane potential/mass ratio in DN4 (and CD4^+^) compared to the other thymocytes subsets (Fig. 1C) could indicate that these cells mainly rely on oxidative metabolism and hence utilize the electrochemical gradient for ATP generation, or that their mitochondrial respiration is inactive. We therefore measured also mitochondrial oxidative capacity in bulk thymocytes and in purified DN3 and DN4 thymocytes. Seahorse analyses of oxygen consumption rates (OCR) and extracellular acidification rates (ECAR) to follow mitochondrial OXPHOS and glycolysis indicated that bulk thymocytes were largely oxidative, with high spare respiratory capacity (SRC) after mitochondrial uncoupling with fluoro-carbonyl cyanide phenylhydrazone (FCCP) (Fig. 1D–F). Low concentrations of etomoxir, which block carnitine palmitoyltransferase 1a and thus mitochondrial fatty acid oxidation [28], did not curtail maximal respiration, SRC, and OCR/ECAR ratio, suggesting that fatty acids are not the preferential fuel for OXPHOS in total thymocytes (Fig. 1D–G). When we increased the granularity of our analysis, we found that both basal and maximal OCR and ECAR were higher in DN3 compared to DN4 cells (Fig. 1H, I). Normalization to initial values showed higher SRC and maximal respiration in DN3 cells (Figs. 1J and S1C), while lower OCR/ECAR ratio and higher maximal glycolytic rate in DN4 cells (Figs. 1K and S1D). These results reveal that the lower mitochondrial membrane potential/mitochondrial mass ratio recorded in DN4 cells does not reflect active mitochondrial respiration, but likely mitochondrial inactivity; moreover, they indicate that the increased mitochondrial mass of DN3 cells is accompanied by higher rates of basal respiration. Finally, the excess SRC recorded in DN3 cells suggests that mitochondria of these immature T cells might be structurally organized to efficiently utilize fuels when maximal respiration is stimulated.

### Conditional *Opa1* deletion impairs thymocyte development and mitochondrial metabolism at β-selection stage

**Fig. 2:**
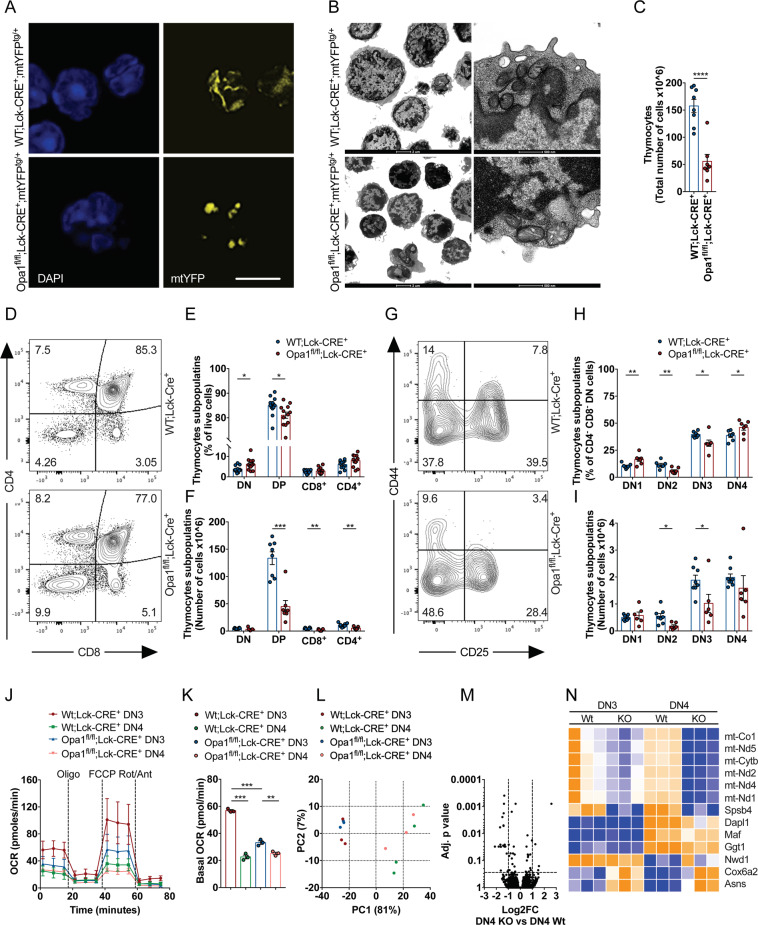
Opa1 deficiency impairs thymocyte development at β-selection stage. **A** Representative confocal images of mtYFP fluorescence in thymocytes freshly isolated from WT;Lck-Cre^+^;mtYFP^+^ and Opa1^fl/fl^;Lck-Cre^+^;mtYFP^+^ mice. Scale bar: 5 µm. **B** Representative electron micrographs of thymocytes freshly isolated from WT;Lck-Cre^+^ and Opa1^fl/fl^;Lck-Cre^+^ mice. Scale bars: 2 µm or 500 nm as indicated. **C** Total thymocyte cellularity of WT;Lck-Cre^+^ and Opa1^fl/fl^;Lck-Cre^+^ mice. Each symbol represents an individual mouse. Data are shown as flow scatter dot plots with mean ± SEM bars (*N* = 7/8 each group). **D** Representative flow cytometry of DN, DP, CD4^+^, CD8^+^ thymocyte subsets in WT;Lck-Cre^+^ and Opa1^fl/fl^;Lck-Cre^+^ mice. **E** Frequencies of DN, DP, CD4^+^, CD8^+^ thymocyte subsets in WT;Lck-Cre^+^ and Opa1^fl/fl^;Lck-Cre^+^ mice. Each dot represents an individual mouse (*N* = 12 each group). Mean ± SEM are reported. **F** Cellularity of DN, DP, CD4^+^, CD8^+^ thymocyte subsets in WT;Lck-Cre^+^ and Opa1^fl/fl^;Lck-Cre^+^ mice. Each dot represents an individual mouse (*N* = 8 each group). Mean ± SEM are reported. **G** Representative flow cytometry of DN1–DN4 subsets (gated on lineage-negative DN thymocytes) in WT;Lck-Cre^+^ and Opa1^fl/fl^;Lck-Cre^+^ mice. **H** Frequencies of DN1–DN4 thymocyte subsets (gated on lineage-negative DN thymocytes) in WT;Lck-Cre^+^ and Opa1^fl/fl^;Lck-Cre^+^ mice. Each dot represents an individual mouse (*N* = 7 each group). Mean ± SEM are reported. **I** Cellularity of DN1–DN4 thymocyte subsets (gated on lineage-negative DN thymocytes) in WT;Lck-Cre^+^ and Opa1^fl/fl^;Lck-Cre^+^ mice. Each dot represents an individual mouse (*N* = 6/8 each group). Mean ± SEM are reported. **J** DN3 and DN4 thymocytes were sorted from WT;Lck-Cre^+^ and Opa1^fl/fl^;Lck-Cre^+^ mice and OCR was measured at baseline and after exposure to oligomycin (Oligo), FCCP, and rotenone/antimycin (Rot/Ant). Data are shown as mean ± SEM (*N* = 4/group). **K** Basal OCR of DN3 and DN4 thymocytes isolated from WT;Lck-Cre^+^ and Opa1^fl/fl^;Lck-Cre^+^ mice. Data are shown as mean ± SEM (*N* = 4/group). **L** Principal component analysis of gene expression of DN3 and DN4 thymocytes isolated from WT;Lck-Cre^+^ and Opa1^fl/fl^;Lck-Cre^+^ mice. **M** Gene expression of DN4 Opa1^fl/fl^;Lck-Cre^+^ versus DN4 WT;Lck-Cre^+^ thymocytes analyzed by RNAseq. **N** Heatmap visualization of the 13 significantly differentially expressed genes between DN4 Opa1^fl/fl^;Lck-Cre^+^ versus DN4 WT;Lck-Cre^+^ thymocytes. *N* = 3/group.

Our metabolic and cytofluorimetric profiling supported a role for mitochondrial respiration at the DN3 stage of pre-T cells. Interestingly, memory T (T_M_) cells display similar metabolic features and deletion of the mitochondria-shaping protein OPA1 not only alters the metabolic signature of T_M_ cells, but it also skews them toward an effector T (T_E_) cell phenotype [24]. We first checked OPA1 levels during the different developmental stages of DN thymocytes. OPA1 expression was sustained throughout DN thymocyte development (Fig. S2A). Given the central role of OPA1 in mitochondrial metabolism and its high expression during T cell development, we sought to investigate if deletion of *Opa1* affected the metabolic and immunological profiles of thymocytes. To ablate *Opa1* from the DN stage of early T cell development, we crossed Opa1^fl/fl^ [23] with Lck-Cre mice. Because Lck-Cre expression can per se affect thymocyte development [29, 30], we always compared Opa1^fl/fl^;Lck-Cre^+^ mice with their littermates expressing only Lck-Cre (hereafter referred to as WT;Lck-Cre^+^, Fig. S2B, C). Efficient reduction of OPA1 levels was confirmed by immunoblotting of thymocyte lysates (Fig. S2D). To study mitochondrial morphology directly ex vivo, we crossed WT and Opa1^fl/fl^;Lck-Cre^+^ mice to a reporter mouse driving expression of YFP targeted to the mitochondrial matrix (mtYFP) in cells where Cre recombinase is active [31]. The offspring (hereafter WT;Lck-Cre^+^;mtYFP^tg/+^ and Opa1^fl/fl^;Lck-Cre^+^;mtYFP^tg/+^ mice) stably expresses mtYFP in thymocytes upon Lck-Cre activation and allows imaging of mitochondria in freshly purified thymocytes. Confocal imaging of mtYFP revealed indeed the expected fragmentation in *Opa1*-deficient cells compared to the WT counterparts (Fig. 2A). Moreover, electron micrographs showed cristae remodeling and disruption upon *Opa1* deletion (Fig. 2B). When we analyzed the immunophenotype of *Opa1*-deficient thymi, we found a threefold reduction in total cellularity (Fig. 2C). Frequencies of DP thymocytes were reduced, whereas the % of DN cells appeared to increase (Fig. 2D, E). In absolute numbers, DP, CD4^+^, and CD8^+^ cells were, however, all reduced (Fig. 2F). This observation led us to hypothesize that *Opa1* deletion during T cell development affected the DN → DP transition. Thus, we analyzed thymic development at the earlier stages of DN T cell progenitors. Frequency and total number of DN2, but especially DN3 cells were reduced in Opa1^fl/fl^;Lck-Cre^+^ mice compared to WT littermates (Fig. 2G–I). Expression of the NOTCH1-dependent gene CD25 was curtailed, possibly reflecting altered IL-2 signaling and *β*-selection [3, 32, 33] (Fig. 2G). To understand whether lack of OPA1 indeed changed the metabolic profiles of T cells undergoing *β*-selection, we sorted DN3 and DN4 cells from WT;Lck-Cre^+^ and Opa1^fl/fl^;Lck-Cre^+^ mice and analyzed their OCR (Fig. 2J). The high basal and maximal respiration observed in WT;Lck-Cre^+^ DN3 cells was lost in Opa1^fl/fl^;Lck-Cre^+^ DN3 cells, indicating that OPA1 expression is crucial for the mitochondrial phenotype of DN3 cells (Fig. 2J, K). The mitochondrial respiration defects observed in *Opa1*-deficient DN3 thymocytes were not a bystander effect of different mitochondrial mass or membrane potential that remained unaltered between WT;Lck-Cre^+^ and Opa1^fl/fl^;Lck-Cre^+^ DN3 and DN4 cells (Fig. S3A, B). Alteration of metabolism during T cell development skews αβ thymocytes into γδ differentiation [13]. However, TCRαβ and TCRγδ populations or expression of the IL-7 receptor CD127 in both total thymocytes (Fig. S4A–D) and in DN cells (Fig. S4E–H) were not affected by *Opa1* deletion.

We next addressed whether OPA1 overexpression affected thymocyte development. To this end, we performed an immune characterization of Opa1^TG^ mice that mildly overexpress OPA1 in all tissues [23]. Thymocyte development both at advanced developmental stage at DP → SP transition as well as early DN3 → DN4 transition and β-selection was not affected (Fig. S5A, B). Similarly, mature T and B cell populations were not altered by OPA1 overexpression (Fig. S5C, D).

Altogether, these data indicate that OPA1 is required for mitochondrial respiration and thymocyte development during *β*-selection.

### *Opa1*-deficient thymocytes are more susceptible to TCR stimulation and apoptosis

To gain insights into the potential mechanism by which *Opa1* deletion impacts *β*-selection, we performed RNA sequencing (RNAseq) analysis of sorted DN3 and DN4 Wt and *Opa1*-deleted T cells. While this approach had been informative to define the role of mitochondrial dynamics in cardiac development and angiogenesis [34, 35], it only revealed mild overall differences between Wt and *Opa1* knockout thymocytes. Indeed, when we performed a principal component analysis on the RNAseq data, we observed a major separation only between DN3 and DN4 subpopulations, irrespective of *Opa1* deletion (Fig. 2L). Gene set enrichment analysis of differentially expressed genes between WT;Lck-Cre^+^ DN3 and DN4 cells showed, as expected, modulation of NOTCH signaling and *Il2ra* (CD25) downregulation during β-selection, confirming cell population identity (Fig. S6). Despite this overall similarity between WT;Lck-Cre^+^ and Opa1^fl/fl^;Lck-Cre^+^ cells, the expression of 13 genes was significantly altered in *Opa1*-deficient cells (Fig. 2M, N). Seven genes were less expressed in *Opa1*-deficient DN3 cells and plummeted even more at the DN4 stage; of these, six were subunits of the ETC encoded by mitochondrial DNA (mtDNA) (Fig. 2N). We were not surprised to find that these genes were less expressed, because long-term *Opa1* deletion impairs mtDNA translation [23]. Interestingly, two genes that are normally expressed at low levels in WT DN3 and even less in DN4 cells were upregulated in both DN3 and DN4 *Opa1*-deficient T cells: *Cox6a2* coding for a respiratory chain subunit that stabilizes dimers of complex IV [36]; and Asparagine synthetase (*Asns*), coding for the enzyme that synthetizes asparagine from aspartate [37] and a relapse gene in early T cell precursor acute lymphoblastic leukemia [38]. Overexpression of *Cox6a2* can reflect a compensatory process that stabilizes respiratory chain supercomplexes, otherwise unstable in *Opa1*^−/−^ cells [23]. Asparaginase upregulation could reflect the attempt to dispose of the aspartate that accumulates because of mitochondrial dysfunction and reduced TCA cycle flux. Indeed, levels of aspartate are reduced in *Opa1*-deficient neurons and in plasma of patients with autosomal dominant optic atrophy caused by *OPA1* mutations [39]. Nevertheless, we failed to find a unique transcriptional signature that could explain why we found less DN3 cells in *Opa1*-deficient thymi. We therefore turned our attention to the immunophenotype of Opa1^fl/fl^;Lck-Cre^+^ DN3 cells.

**Fig. 3:**
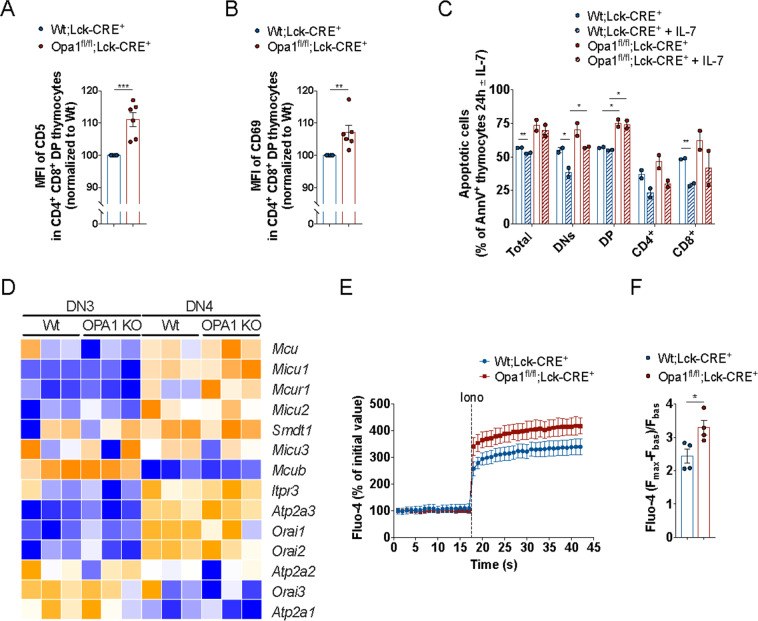
Opa1 deficiency alters TCR and calcium signaling with increased susceptibility to apoptosis. **A** MFI of CD5 in WT;Lck-Cre^+^ and Opa1^fl/fl^;Lck-Cre^+^ analyzed by flow cytometry. Data are mean ± SEM (*N* = 6). **B** MFI of CD69 in WT;Lck-Cre^+^ and Opa1^fl/fl^;Lck-Cre^+^ analyzed by flow cytometry. Data are mean ± SEM (*N* = 6). **C** Frequencies of apoptotic cells (AnnV^+^ cells) in WT;Lck-Cre^+^ and Opa1^fl/fl^;Lck-Cre^+^ measured by flow cytometry 24 h after isolation and culture ± IL-7. Data are mean ± SEM (*N* = 2). **D** Heatmap visualization of genes encoding for mitochondrial and cytoplasmic calcium handling regulators in DN3 and DN4 Opa1^fl/fl^;Lck-Cre^+^ versus WT;Lck-Cre^+^ thymocytes (*N* = 3/group). **E** Cytoplasmic calcium levels at baseline and after Ionomycin (Iono) in Opa1^fl/fl^;Lck-Cre^+^ versus WT;Lck-Cre^+^ thymocytes. Data are mean ± SEM (*N* = 4). **F** Maximal cytoplasmic calcium level in Opa1^fl/fl^;Lck-Cre^+^ versus WT;Lck-Cre^+^ thymocytes. Data are mean ± SEM (*N* = 4).

Lower CD25 expression in Opa1^fl/fl^;Lck-Cre^+^ DN3 cells (Fig. 2G) could reflect different TCR signaling strength and thus a differential activation status of WT;Lck-Cre^+^ and Opa1^fl/fl^;Lck-Cre^+^ thymocytes [3, 40]. Because upregulation of CD5 and CD69 during antigen stimulation positively correlates with the strength of the TCR signaling [41, 42], we analyzed their expression levels. Levels of both CD5 and CD69 were higher in DP Opa1^fl/fl^;Lck-Cre^+^ thymocytes, suggesting stronger TCR signaling in *Opa1*-deficient T cells (Fig. 3A, B). TCR engagement leads to endoplasmic reticulum Ca^2+^ release and CRAC channels opening on the cell membrane [43], ultimately resulting in Ca^2+^-dependent cell death [44]. Although immediately after isolation levels of apoptosis were not different in WT;Lck-Cre^+^ and Opa1^fl/fl^;Lck-Cre^+^ thymocytes (Fig. S7A), *Opa1*-deficient T cells were more sensitive to spontaneous cell death after 24 h in culture (Fig. 3C). Supplementation with IL-7 did not differentially rescue Wt or *Opa1*-deficient cells from apoptosis (Fig. 3C), in line with the comparable expression of CD127 (IL-7 receptor) in WT;Lck-Cre^+^ and Opa1^fl/fl^;Lck-Cre^+^ thymocytes (Fig. S7B, C). Because our data pointed to a role for Ca^2+^ signaling defects upon *Opa1* deletion, a phenotype observed also in other tissues [45], we decided to further investigate this crucial aspect of AICD. We took a two-pronged approach and analyzed both gene expression of molecules involved in Ca^2+^ signaling as well as the dynamics of Ca^2+^ signaling in Wt and *Opa1*-deficient T cells.

Using our RNAseq data set, we delved into the expression of genes encoding for different regulators of intracellular calcium signaling: IP3R, SERCA, ORAI channels, and the mitochondrial calcium uniporter (MCU). The MCU holoplex consists of the MCU ion pore, its negative regulatory subunit MCUb, the essential regulator Smdt1/EMRE, the two regulatory subunits MICU1 and MICU2 (and the CNS paralogue MICU3), and the MICUR1 regulator [46]. This analysis showed Ca^2+^ genes were upregulated in the DN3–DN4 transition, and that deletion of O*pa1* had no effect on the expression of these studied genes. Interestingly, the DN3 → DN4 transition appears to be crucial to enable mitochondrial Ca^2+^ uptake: while the core MCU holoplex components are all upregulated, the inhibitory MCU subunit MCUb is downregulated. Despite we did not find a “Ca^2+^ signaling signature” associated with *Opa1* deletion, our previous studies showed that lack of *Opa1* affects Ca^2+^ signaling by modifying the supramolecular organization of the MCU holoplex [45]. We therefore performed a set of functional Ca^2+^ measurements in ex vivo thymocytes and in Jurkat cells. In thymocytes challenged with ionomycin to evaluate maximal cytoplasmic calcium levels by Fluo-4 staining, we observed that maximal cytoplasmic Ca^2+^ levels where higher in *Opa1*-deficient cells than in their WT counterparts. To evaluate the specific effect of OPA1 inhibition and deletion on intracellular Ca^2+^ changes downstream TCR activation, we turned to Jurkat cells that represent the standard in vitro model for TCR signaling studies. To inhibit Opa1, we treated Jurkat cells with the specific Opa1 inhibitor MYLS22 [35] and we downregulated *OPA1* expression by siRNA. Both inhibition and downregulation of OPA1 resulted in higher maximal and residual cytoplasmic calcium levels (Fig. S8A, B). Overall, these data suggest that *Opa1* deficiency alters TCR signaling in thymocytes, possibly contributing to their higher susceptibility to apoptosis.

### Lymphopenia and functional defects in mature T cells from *Opa1* thymocyte knockout mice

**Fig. 4:**
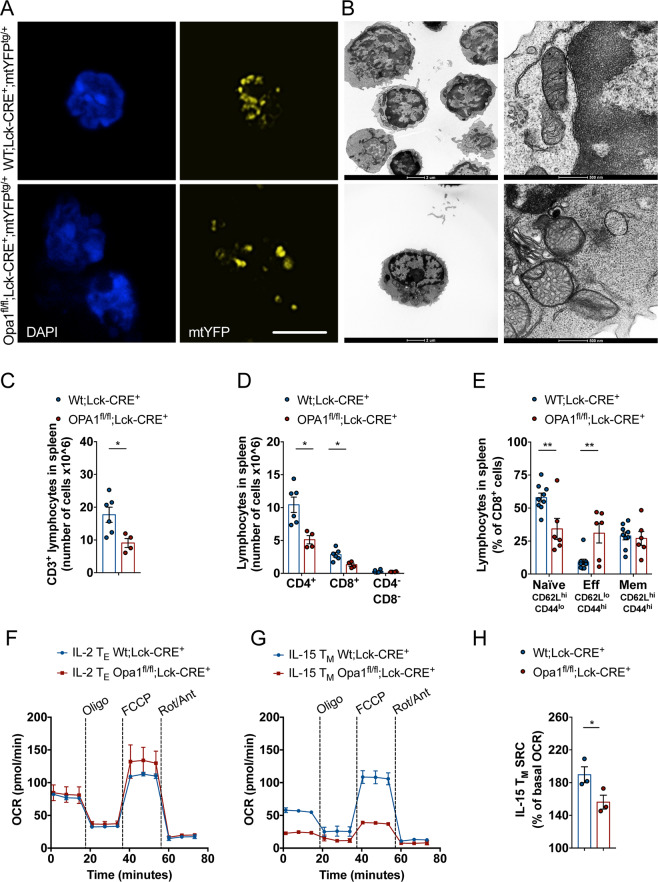
Opa1 deficiency generates structurally, functionally, and metabolically impaired mature CD8^+^ T cells. **A** Representative confocal images of mtYFP fluorescence in CD8^+^ lymphocytes freshly isolated from WT;Lck-Cre^+^;mtYFP^+^ and Opa1^fl/fl^;Lck-Cre^+^;mtYFP^+^ mice. Scale bar: 5 µm. **B** Representative electron micrographs of CD8^+^ lymphocytes freshly isolated from WT;Lck-Cre^+^ and Opa1^fl/fl^;Lck-Cre^+^ mice. Scale bars: 2 µm or 500 nm as indicated. **C** Cellularity of CD3^+^ splenocytes in WT;Lck-Cre^+^ and Opa1^fl/fl^;Lck-Cre^+^ mice. Each symbol represents an individual mouse. Each dot represents an individual mouse (*N* = 4/6 each group). Mean ± SEM are reported. **D** Cellularity of CD4^+^, CD8^+^, and CD4^−^ CD8^−^ splenocytes in WT;Lck-Cre^+^ and Opa1^fl/fl^;Lck-Cre^+^ mice. Each dot represents an individual mouse (*N* = 4/6 each group). Mean ± SEM are reported. **E** Frequencies of naive, effector, and memory CD8+ T lymphocytes in WT;Lck-Cre^+^ and Opa1^fl/fl^;Lck-Cre^+^ mice. Each dot represents an individual mouse (*N* = 6/9 each group). Mean ± SEM are reported. **F** OCR of WT;Lck-Cre^+^ and Opa1^fl/fl^;Lck-Cre^+^ CD8^+^ T cells activated with αCD3/αCD28 and differentiated in IL-2 T_E_ cells for 6 days. OCR analysis at baseline and after exposure to oligomycin (Oligo), FCCP, and rotenone/antimycin (Rot/Ant). Data are representative of three independent experiments (*N* = 3/group). **G** OCR of WT;Lck-Cre^+^ and Opa1^fl/fl^;Lck-Cre^+^ CD8^+^ T cells activated with αCD3/αCD28 and differentiated in IL-15 T_M_ cells for 6 days. OCR analysis at baseline and after exposure to oligomycin (Oligo), FCCP, and rotenone/antimycin (Rot/Ant). Data are representative of three independent experiments (*N* = 3/group). **H** SRC of WT;Lck-Cre^+^ and Opa1^fl/fl^;Lck-Cre^+^ CD8^+^ T cells treated as in (**F**). Data are mean ± SEM (*N* = 3).

To verify if the alteration in T cell development observed upon early *Opa1* deletion reverberated in mature T cells, we first analyzed the hematological profile of WT;Lck-Cre^+^ and Opa1^fl/fl^;Lck-Cre^+^ mice. Total blood count analysis showed no differences between knockout and WT animals (Fig. S9A). We delved further in the mature T cell compartment, first by analyzing mitochondrial morphology and ultrastructure in CD8^+^ lymphocytes sorted from spleens. We used WT;Lck-Cre^+^;mtYFP^tg/+^ and Opa1^fl/fl^;Lck-Cre^+^;mtYFP^tg/+^ mice to identify and sort Lck-Cre^+^ T cells from spleens and to directly inspect mitochondrial morphology. Mitochondria were fragmented (Fig. 4A) and cristae swollen (Fig. 4B) in *Opa1*-deficient, mtYFP^+^, CD8^+^ T cells compared to their mtYFP^+^ WT counterparts. T cell function, activation, and differentiation are largely controlled by specific metabolic requirements: naïve and memory cells rely on OXPHOS, whereas activated effector cells are metabolically active, mostly depending on glycolysis for ATP generation [47]. Given the profound mitochondrial changes recorded in *Opa1*^−/−^, mtYFP^+^, CD8^+^ T cells, we compared T cell differentiation and activation in WT and *Opa1*^−/−^ CD8^+^ T cells. Total T cells (CD3^+^) as well as CD4^+^ and CD8^+^ T cells subsets were reduced in Opa1^fl/fl^;Lck-Cre^+^;mtYFP^tg/+^ mice (Fig. 4C, D), suggesting the development of lymphopenia. Interestingly, while CD4^+^ and CD8^+^ relative abundance was not altered in Opa1^fl/fl^;Lck-Cre^+^;mtYFP^tg/+^ mice (Fig. S9B, C), the frequency of CD3^+^CD4^−^CD8^−^ T (DNT) cells was increased (Fig. S9C–E). These cells represent an immunoregulatory T cell subset, sharing some features and surface markers with CD4 Treg and NK cells [48] and frequently expressing γδ chains. Furthermore, we observed a reduction of Naïve (CD62L^high^CD44^low^) CD8^+^ T cells and the accumulation of effector memory (CD62L^low^CD44^high^) CD8^+^ T cells in homeostatic condition (i.e., without any infection) in Opa1^fl/fl^;Lck-Cre^+^ mice (Fig. 4E). This immunophenotype could mirror the activated phenotype of thymocytes during maturation, characterized by the CD5 and CD69 upregulation in DP thymocytes (Fig. 3A, B). The accumulation of effector memory T cells in homeostatic conditions was not surprising, given that OPA1 is dispensable for T_E_ cells development [24]. We further verified if *Opa1* deletion during early thymocyte development affected the generation and the metabolism of T_M_ and T_E_ cells generated in vitro by treating CD8^+^ T cells with IL-15 and IL-2, respectively. The metabolism of T_E_ cells was not affected by *Opa1* deficiency (Fig. 4F). Indeed, OXPHOS defects were not apparent in in vitro generated *Opa1*-deficient T_E_ cells (Fig. 4F), in line with the glycolytic phenotype of these cells [24]. Conversely, in *Opa1*^−/−^ T_M_ generated in vitro the basal OCR was reduced and the high SRC typical of WT T_M_ was abolished (Fig. 4G, H). Thus, OPA1 is essential to generate metabolically fit T_M_. In sum, the absence of *Opa1* during thymocyte development results in mitochondrial, functional, and metabolic defects affecting mature T cells in peripheral lymphoid tissues.

## Discussion

Despite the emerging role of metabolism in the immune response [47], the question of whether and how mitochondrial dynamics and OPA1 specifically direct T cell development remains unanswered. Here, we found that DN3 T cell progenitors predominantly rely on OXPHOS over glycolysis and that OPA1 is required for *β*-selection and generation of metabolically competent mature lymphocytes.

Thymic development is particularly sensitive to perturbation of mitochondrial content and function [11–13, 49–51]. Mitochondria are less abundant in mature T cells than thymocytes and are cleared by increased levels of autophagy during their development [52, 53]. Of note, mitochondrial dynamics and autophagy crosstalk in the regulation of AICD [26, 27], which suggests that unbalanced mitochondria quality control might result in lymphocyte functional deficiencies or autoimmunity. Our data show how the absence of OPA1, a master regulator of IMM fusion and ETC supercomplexes organization, hijacks thymocyte metabolism and development starting from *β*-selection stage.

NOTCH signaling is critical during thymocyte maturation. It promotes survival of pre-T cells at the β-selection checkpoint [5, 10, 11] and together with IL-7 and mTORC signaling determines proliferation and T cell lineage commitment [6, 13, 54]. Interestingly, mitochondrial fusion directs the differentiation of cardiomyocytes via Calcineurin and NOTCH signaling [55]. Our current data suggest the crosstalk between mitochondrial shape and NOTCH signaling might also extend to thymocyte development.

Thymocyte maturation requires coordinated and controlled cell movement and migration in the different areas of the thymus, which consists anatomically of two interconnected lobes divided in a cortical area (cortex) and inner area (medulla) [56]. T cell progenitors, after entering the organ, migrate toward the cortex where they progress in maturation up to DP stage before reaching the medulla to complete negative selection and single positive T cell commitment [57]. Mitochondrial fragmentation and relocation to the uropod (the posterior area) of a migrating cell is necessary to allow proper chemotaxis and migration [18]. DRP1 ablation results in altered in vitro and in vivo thymocyte migration in the medullary area [20]. It is tempting to speculate that a similar migratory defect might affect *Opa1*-deficient cells. Of note, the higher expression of CD5 and CD69 in DP thymocytes might indeed also reflect longer persistence in the cortex due to a cell migration deficit.

*Opa1* deficiency results in impaired long-term immune protection due to the bioenergetic failure of memory T cells to sustain their viability [24, 47]. Our data show that *Opa1* deficiency starting from early thymocyte development results in lymphopenia and metabolic defects in mature T cells. Moreover, our data suggest that the absence of OPA1 might result in the generation of abnormally activated T cells, possibly contributing to inflammation in a mechanism similar to the one observed for other mitochondrial functional deficiencies [58–60].

Interestingly, mice with T cell specific deletion of OPA1 are lymphopenic but accumulate the immunoregulatory population of CD3^+^CD4^−^CD8^−^ DNT cells predominantly expressing TCRγδ. This phenotype resembles one of the pathologic features of aplastic anemia [61], a condition where the insufficient number of hematopoietic cells produced in the bone marrow leads to higher susceptibility to infections. Nevertheless, whether human CD3^+^CD4^−^CD8^−^ DNT show a similar mitochondrial bioenergetic defect and whether a genetic or pharmacological insult to mitochondria in the hematopoietic compartment might contribute to the development of aplastic anemia are not known.

While *Opa1* deletion impairs mitochondrial respiration and β-selection in thymocytes, OPA1 overexpression does not affect T cell development. It remains to be investigated whether OPA1 overexpression confers in vivo survival or metabolic advantage which might, on one side, favor the generation of metabolically fit pool of long-lived memory T cells while, on the other side, be detrimental in autoimmune condition where cell death is central to control and limit T cell-mediated tissue damage.

In conclusion, the deletion of *Opa1* early in the T cell lineage hampers mitochondrial respiration at the DN3 stage and β-selection. Moreover, *Opa1* deficiency leads to abnormally activated mature T cells that might possess a pro-inflammatory phenotype.

## Material and methods

### Mice

*Opa1*^fl/fl^ mice are described elsewhere [23]. Mito-YFP mice were a kind gift from Nils-Göran Larsson (Karolinska Institute, University of Stockholm) [31]. Lck-CRE mice (strain ID 003802) were purchased form Jackson lab. Mice were maintained in C57BL6 background and used for experiments between 6 and 8 weeks of age. All mice were bred and maintained under approved protocols (protocol 32/2011 CEASA and 318/2015 Italian Ministry of Health to LS).

### Cell culture and treatments

Jurkat cells were cultured in 1640 media supplemented with 10% FCS, 4 mM L-glutamine, 1% penicillin/streptomycin, and 55 μM β-mercaptoethanol under 5% CO_2_, atmospheric oxygen, at 37 °C in a humidified incubator. For MYLS22 treatment, cells were washed and resuspended in RPMI supplemented with 2% FCS and 50 μM MYLS22 for 2 h before the experiment was started. The following siRNA have been used: Control Silencer siRNA (4390843) and *OPA1* silencer siRNA (4392420 ID s9850) from ThermoFisher. Jurkat cells were electroporated with 100 nM siRNA using Neon Transfection System (Invitrogen) following manufacturer’s protocol. Cells were analyzed 24 h after electroporation.

### Flow cytometry

Fluorochrome-conjugated monoclonal antibodies were purchased from eBioscience, BD Pharmingen, or Biolegend. Single cell suspension were prepared form thymus or spleens of WT;Lck-Cre^+^ and Opa1^fl/fl^;Lck-Cre^+^ mice. Erythrocytes were eliminated by 10 min incubated with ACK buffer (ThermoFisher). Equal number of cells were stained with indicated antibodies diluted 1:200 in PBS supplemented with 1% BSA for 20 min at 4 °C. MitoTracker Green and TMRM staining were performed according to the manufacturer’s instructions (Life Technologies). Equal number of thymocytes were loaded with 15 nM MitoTracker Green or 2 nM TMRM for 30 min at 37 °C in humidified incubator. CsH (0.5 µM) was added to each sample to inhibit the multidrug resistance pump. Acquisition and analysis were performed using a FACSCanto^TM^ II and FlowJo software (BD Biosciences).

### Confocal imaging

Mito-YFP^+^ cells from WT;Lck-Cre^+^ and Opa1^fl/fl^;Lck-Cre^+^ mice were sorted using a FACSAria^TM^ Illu Cell Sorter (BD Biosciences) and fixed on poly-d-lysine pre-coated slides. For confocal images, microscope slides were placed on the stage of a LSM510 Zeiss confocal imaging system using a Plan Apochromat ×63 1.4 NA Oil DIC objective or on a Nikon A1r microscope using a ×40 CFI Plan APO VC 1.4 NA DT:0.13 mm objective. Stacks of images separated by 0.4 μm along the *z*‐axis were acquired. Then, three‐dimensional reconstruction of the mitochondrial stacks was performed with ImageJ 1.44o (NIH).

### Electron microscopy

T cells (2 × 10^6^) were fixed in 2.5% glutaraldehyde in 100 mM sodium cacodylate, washed in cacodylate buffer. After dehydration samples were embedded in Eponate 12 resin (Ted Pella) and sections were cut. Images were acquired using a FEI Tecnai 12 Transmission electron microscope equipped with a Veleta digital camera. Brightness and contrast were adjusted using ImageJ 1.44o (NIH).

### Western blotting

Cells were washed with ice cold PBS and lysed in 1x lysis buffer (Cell Signaling Technologies) supplemented with 1 mM PMSF and protease inhibitors. Samples were incubated 30 min on ice before being centrifuged at 20,000 × *g* for 10 min at 4 °C. Cleared protein lysate was denatured with LDS loading buffer for 10 min at 90 °C. Samples were run on precast 4–12% bis-tris protein gels (Life Technologies). Proteins were transferred onto nitrocellulose membranes using an iBLOT 2 system (Life Technologies). Membranes were blocked with 5% w/v milk and 0.1% Tween-20 in TBS and incubated with the appropriate antibodies in 5% w/v BSA in TBS with 0.1% Tween-20 overnight at 4 °C. The following antibodies were used: anti-OPA1 (BD Biosciences, Cat# 612606) and anti-Actin (CST, Cat# 4970S). All primary antibody incubations were followed by incubation with secondary HRP-conjugated antibody (Pierce) in 5% milk and 0.1% Tween-20 in TBS and visualized using SuperSignal West Pico or Femto Chemiluminescent Substrate (Pierce).

### Metabolic phenotyping

OCR and ECAR were measured in XF media (non-buffered RPMI 1640 containing 25 mM glucose, 2 mM L-glutamine, and 1 mM sodium pyruvate) under basal conditions and in response to 3 μM etomoxir (Tocris), 1 μM oligomycin, 1.5 μM FCCP, and 100 nM rotenone + 1 μM antimycin A, using a 96 well XF or XFe extracellular flux analyzer (Seahorse Bioscience).

### Calcium measurements

Thymocytes (3 × 10^6^) were loaded for 30 min at 37 °C in humidified incubator with 6 μM Fluo-4 dissolved in HBSS supplemented with calcium and magnesium (Sigma H8264) in the presence of 0.5 µM CsH. Cells were then washed and resuspended in 300 μl of HBSS + calcium and magnesium supplemented with 0.5 µM CsH. Fluo-4 fluorescence was acquired using the AF488 channel of the cytofluorimeter. We acquired the basal fluorescence for 30 s followed by Ionomycin treatment (final concentration 500 ng/mL) to evaluate maximal calcium flux.

Jurkat cells (3 × 10^6^) were loaded 30 min at 37 °C in a humidified incubator with 2 µM Indo-AM dissolved in complete medium supplemented with 0.5 µM CsH. Cells were washed and resuspended in 300 μl of complete medium supplemented with 0.5 µM CsH and 5 μg/ml mouse antihuman CD3 (clone OKT3, Invitrogen Cat#16-0037-85) for 30 min at 4 °C in the dark. Indo-violet and indo-blue were acquired in the linear scale of the cytofluorimeter. Tubes were placed in a 37 °C temperature-controlled tube holder and after 45 s acquisition was initiated. After 30 s of basal fluorescence recordings, antiCD3 crosslinking was performed by incubating for 6 min with an anti-mouse IgG (Fc-specific) biotin crosslinking antibody (Sigma, B7401, diluted 1:50 directly in the acquisition tube).

### Complete blood count

Blood from WT;Lck-Cre^+^ and Opa1^fl/fl^;Lck-Cre^+^ mice was collected in heparin coated tubes and analyzed with a CELL-Dyn Emerald hematology analyzer (Abbott, IL, USA).

### RNAseq

RNA isolation was performed using the RNeasy kit (Qiagen) as per manufacturer’s instructions and RNA was quantified using Qubit 2.0 (ThermoFisher Scientific). Libraries were prepared using the TruSeq stranded mRNA kit (Illumina) and sequenced in a HISeq 3000 (Illumina) by the Deep-Sequencing Facility at the Max Planck Institute for Immunobiology and Epigenetics. Sequenced libraries were processed in an in-house developed RNAseq pipeline [62] that employs deepTools for Quality control [63, 64], Cutadapt for trimming [65], STAR [66] for mapping and featureCounts [67] to quantify mapped reads. Raw counts of mapped reads were processed in R (Lucent Technologies) with DESeq2 [68] to determine differentially expressed genes and generate normalized read counts to visualize as heatmaps using Morpheus (Broad Institute).

### Statistical analysis

Flow cytometry data were analyzed using FlowJo 10 (BD Biosciences). Statistical analysis was performed using Prism 7 software (GraphPad) and results are represented as Mean ± SEM. Individual independent experiments are shown as dots overlaid on the interval graphs. All the acquisitions of the experiments have been performed blinded without knowing the specific condition of each sample. Comparisons between two groups were calculated using unpaired two-tailed Student’s *t* tests. Comparisons among more than two groups were calculated using one-way ANOVA with Bonferroni’s multiple comparison tests. We observed normal distribution and no difference in variance between groups in individual comparisons. Selection of sample size was based on extensive experience with metabolic assays. *p < 0.05, **p < 0.01, ***p < 0.001.

## Supplementary information

Supplementary online material

## Data Availability

RNAseq data are accessible at GEO under the accession number GSE165579. The other data sets used and/or analyzed during the current study are available from the corresponding author on reasonable request.
