# Supplementary material for: Deletion of the mitochondria-shaping protein Opa1 during early thymocyte maturation impacts mature memory T cell metabolism
Source: Cell Death Differ. 2021 Mar 1;28(7):2194–206. doi: 10.1038/s41418-021-00747-6 (PMC8257785; doi:10.1038/s41418-021-00747-6)
Supplement: Supplementary file 1 — Supplementary online material [file 41418_2021_747_MOESM1_ESM.docx]

**Deletion of the mitochondria-shaping protein *Opa1* during early thymocyte maturation impacts on mature memory T cells metabolism.**

Corrado et al

**Supplementary material**

**Supplementary online Figures**


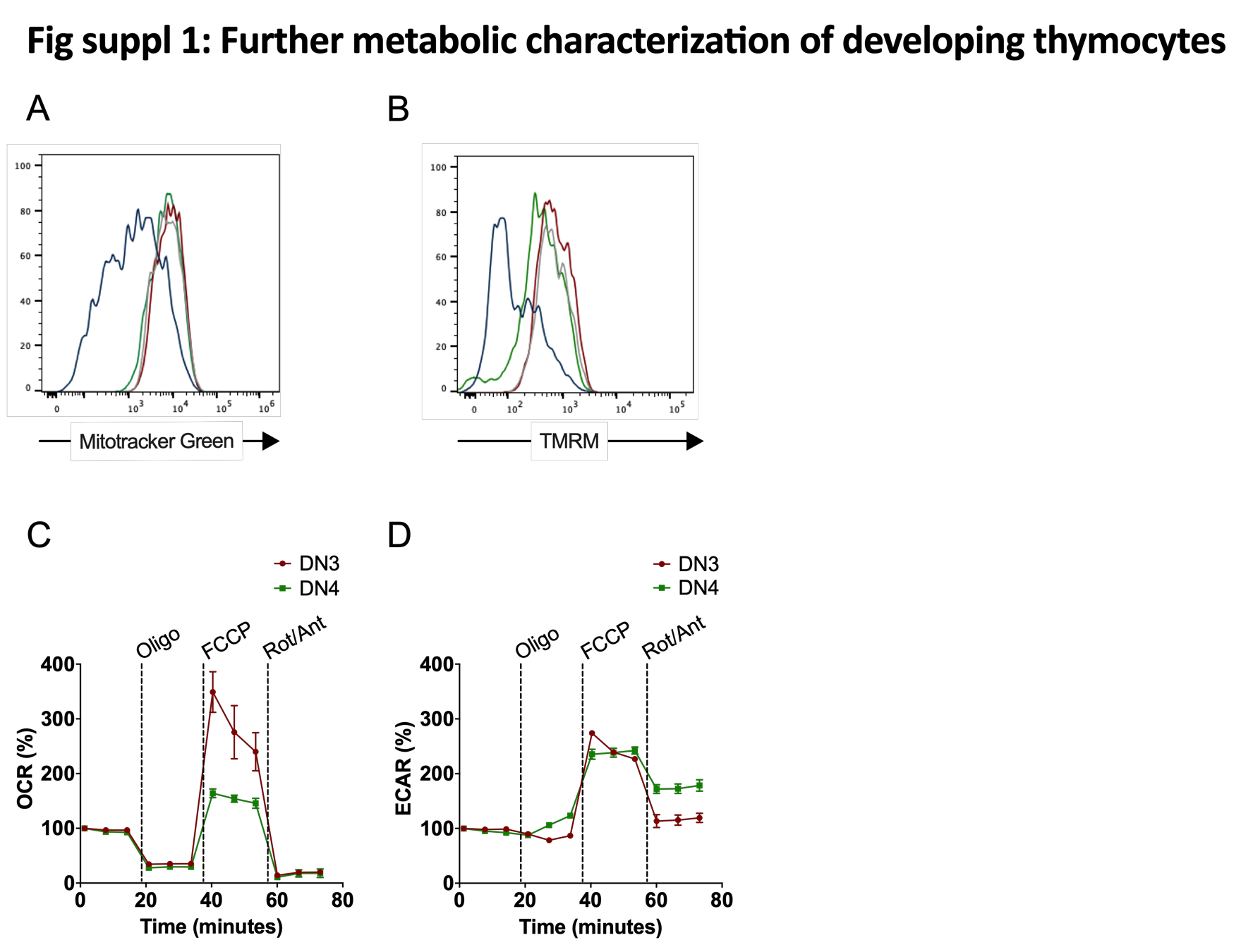


**Supplementary Figure 1: Further metabolic characterization of developing thymocytes.**

1. Representative flow cytometry plot of mitochondrial mass measured with MitoTracker Green in DN3 and DN4 WT;Lck-Cre^+^ thymocytes.
2. Representative flow cytometry plot of mitochondrial membrane potential measured with TMRM in DN3 and DN4 WT;Lck-Cre^+^ thymocytes.
3. OCR normalized (over initial value) of sorted DN3 and DN4 thymocytes. Data are shown as mean ± SEM (N=3)
4. ECAR normalized (over initial value) of sorted DN3 and DN4 thymocytes. Data are shown as mean ± SEM (N=3)


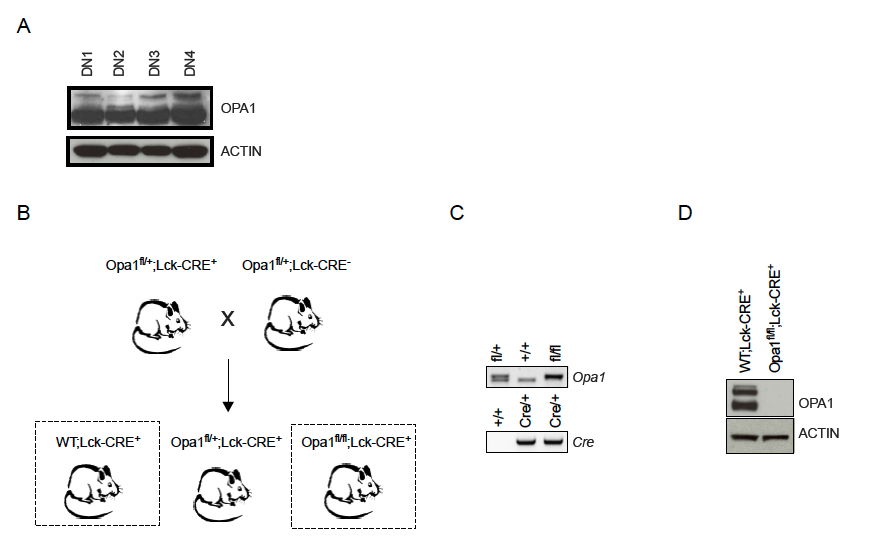


**Supplementary Figure 2: Generation and validation of a T cell specific *Opa1* knockout mouse.**

1. DN thymocytes were sorted according to surface markers form WT mice. . Protein lysates were prepared and equal amounts of proteins were separated by SDS-PAGE and immunoblotted using the indicated antibodies.
2. Cartoon of the strategy to generate T cells conditionally deleted for Opa1. Opa1 mice were crossed to Lck-Cre transgenic mice as indicated. Boxes indicate mouse genotypes analyzed here
3. Presence of floxed alleles and Lck-Cre recombinase in tissue biopsies from WT;Lck-Cre+ and Opa1fl/fl;Lck-Cre+ mice analyzed by PCR.
4. Thymocytes were isolated from mice of the indicated genotype. Protein lysates were prepared and equal amounts of proteins were separated by SDS-PAGE and immunoblotted using the indicated antibodies.


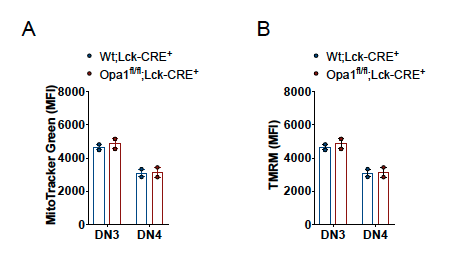


**Supplementary Figure 3: Mitochondrial mass and membrane potential in DN3 and DN4 Wt and *Opa1*^-/-^ thymocytes.**

1. MitoTracker Green staining in DN3 and DN4 Wt and OPA1 KO thymocytes. Data are shown as mean ± SD.
2. TMRM staining in DN3 and DN4 Wt and OPA1 KO thymocytes. Data are shown as mean ± SD.


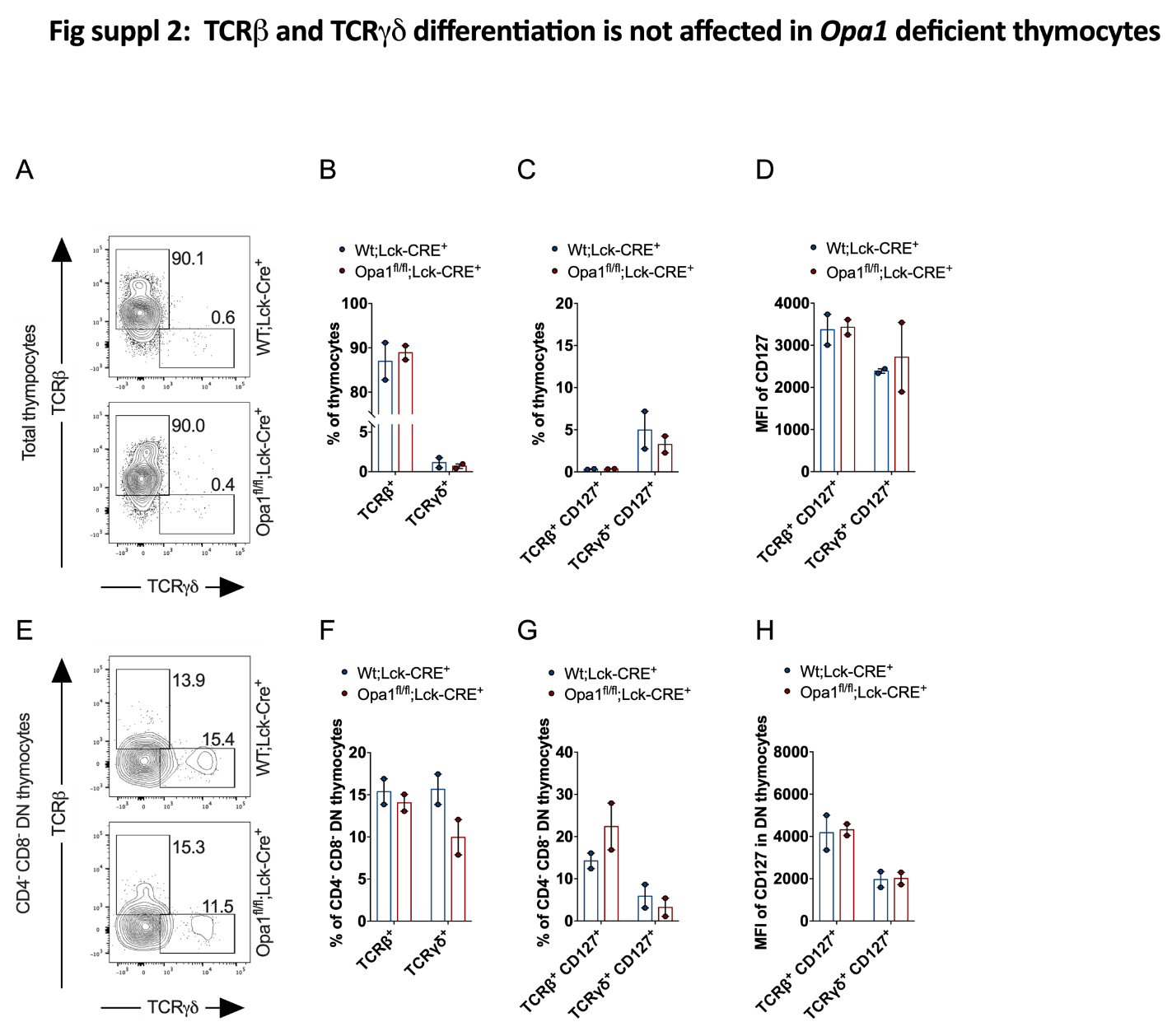


**Supplementary Figure 4: TCRβ and TCRγδ differentiation is not affected in *Opa1* deficient thymocytes.**

1. Representative flow cytometry of TCRβ and TCRγδ subsets in total thymocytes isolated from mice of the indicated genotype.
2. Frequencies of TCRβ and TCRγδ subsets in total thymocytes isolated from mice of the indicated genotype. Data are mean ± SD.
3. Frequencies of TCRβ^+^ CD127^+^ and TCRγδ^+^ CD127^+^ subsets in total thymocytes isolated from mice of the indicated genotype. Data are mean ± SD.
4. Expression of CD127 in TCRβ and TCRγδ subsets in total thymocytes isolated from mice of the indicated genotype. Data are mean ± SD.
5. Representative flow cytometry of TCRβ and TCRγδ subsets in lineage-negative DN thymocytes isolated from mice of the indicated genotype.
6. Frequencies of TCRβ and TCRγδ subsets in lineage-negative DN thymocytes isolated from mice of the indicated genotype. Data are mean ± SD.
7. Frequencies of TCRβ^+^ CD127^+^ and TCRγδ^+^ CD127^+^ subsets in lineage-negative DN thymocytes isolated from mice of the indicated genotype. Data are mean ± SD.
8. Expression of CD127 in TCRβ and TCRγδ subsets in lineage-negative DN thymocytes isolated from mice of the indicated genotype. Data are mean ± SD.

**
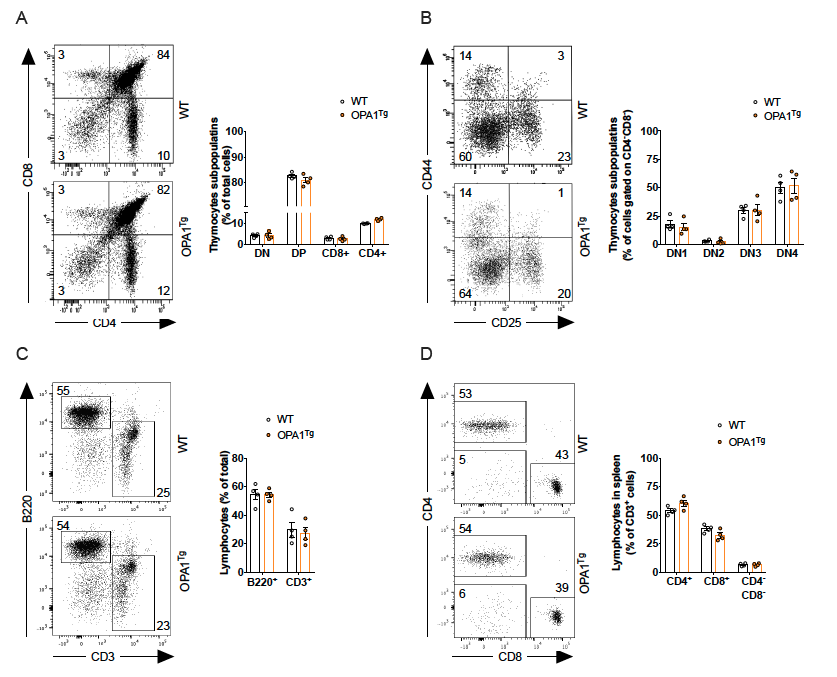
**

**Supplementary Figure 5: Opa1 overexpression does not alter thymocyte development and mature T and B cell populations.**

1. Representative flow cytometry (left) and frequency graph (right) of DN, DP, CD4+, CD8+ thymocyte subsets in mice of the indicated genotype. In the graph, each dot represents an individual mouse (N=3). Data are mean ± SEM.
2. Representative flow cytometry (left) and frequency graph (right) of DN1 to DN4 subsets (gated on lineage-negative DN thymocytes) in in mice of the indicated genotype. In the graph, each dot represents an individual mouse (N=3). Data are mean ± SEM.
3. Representative flow cytometry (left) and frequency graph (right) of B and T cells in mice of the indicated genotype. In the graph, each dot represents an individual mouse (N=3). Data are mean ± SEM.
4. Representative flow cytometry (left) and frequency graph (right) of CD4+ and CD8+ T cells in mice of the indicated genotype. In the graph, each dot represents an individual mouse (N=3). Data are mean ± SEM.


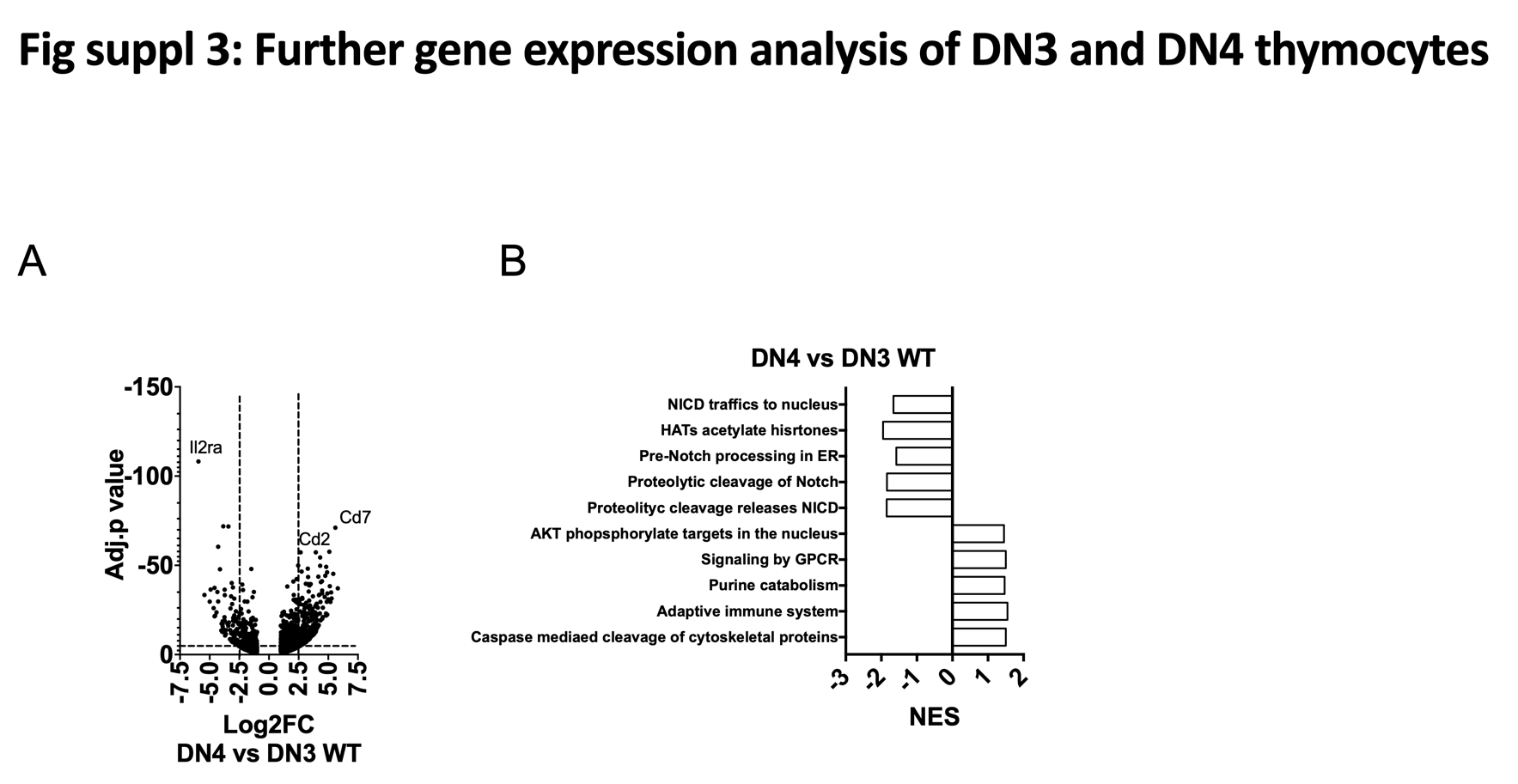


A

B

**Supplementary Figure 6. Gene expression analysis of DN3 and DN4 thymocytes.**

1. Volcano Plot of gene expression in DN4 versus DN3 WT;Lck-Cre^+^ thymocytes analyzed by RNAseq.
2. GSEA analysis of DN4 versus DN3 WT;Lck-Cre^+^ thymocytes analyzed by RNAseq.


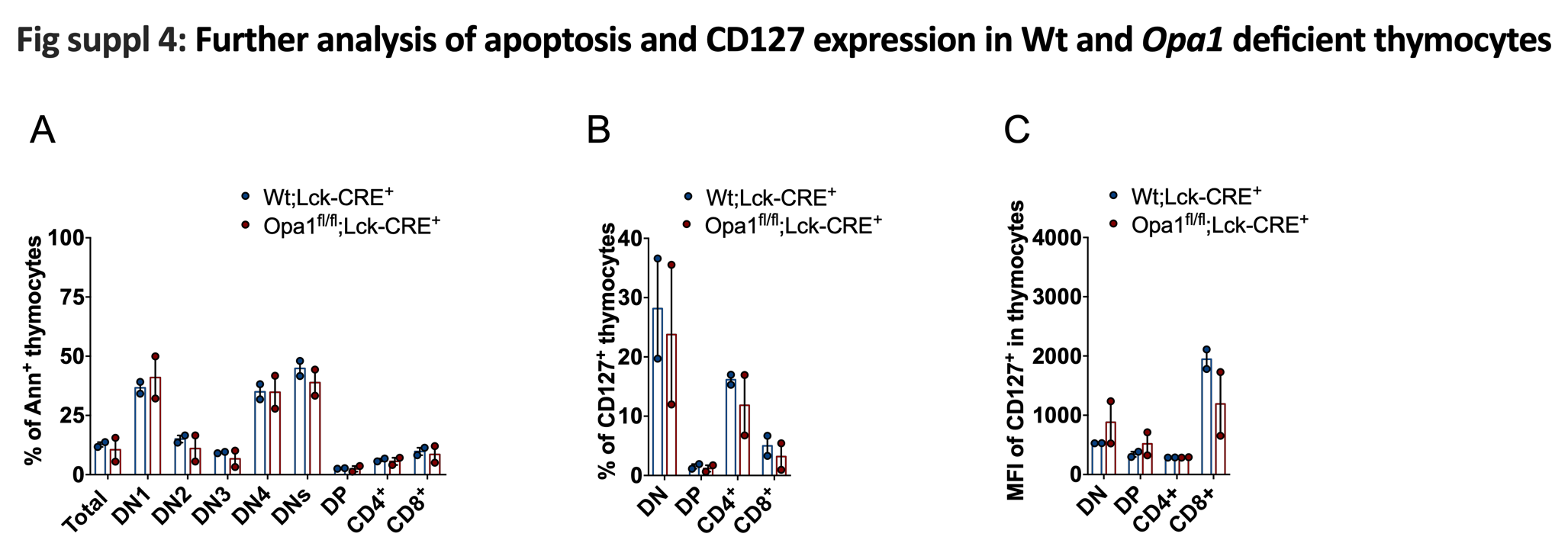


**Supplementary Figure 7: Further analysis of apoptosis and CD127 expression in Wt and *Opa1* deficient thymocytes.**

1. Frequencies of apoptotic cells (measured by flow cytometry as Annexin-V^+^ cells) in freshly isolated thymocytes from littermates of the indicated genotype. Each dot indicates an individual mouse. Data are mean ± SD.
2. Frequencies of CD127^+^ thymocytes isolated from littermates of the indicated genotype. Each dot indicates an individual mouse. Data are mean ± SD.
3. Expression of CD127 in thymocytes isolated from littermates of the indicated genotype. Each dot indicates an individual mouse. Data are mean ± SD.

**
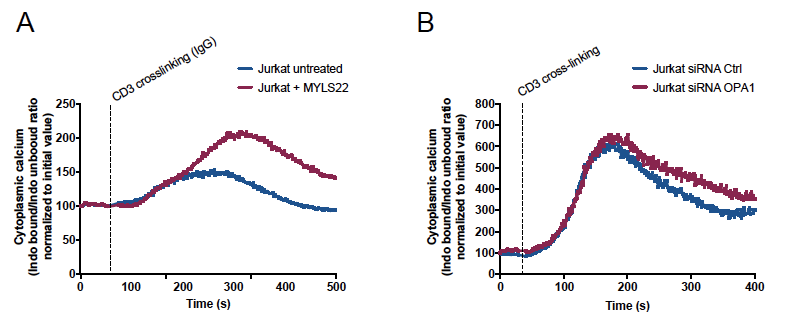
**

**Supplementary Figure 8: Pharmacological inhibition or silencing of OPA1 increases cytoplasmic Ca^2+^ levels following TCR stimulation.**

1. Traces of Indo1 flow cytometric recordings in Jurkat T cells treated with an anti-CD3 antibody. Where indicated, cells were pre-incubated for 2h with of the specific OPA1 inhibitor MYLS22 (50μM). Hatched line indicates anti-CD3 cross-linking and TCR triggering. Data are mean of N=3 independent experiments normalized to the initial value.
2. Experiments were as in (A) except that 24 h before the experiments, cells were electroporated with the indicated siRNA (Ctrl: control unrelated siRNA). Data are mean of N=3 independent experiments normalized to the initial value.


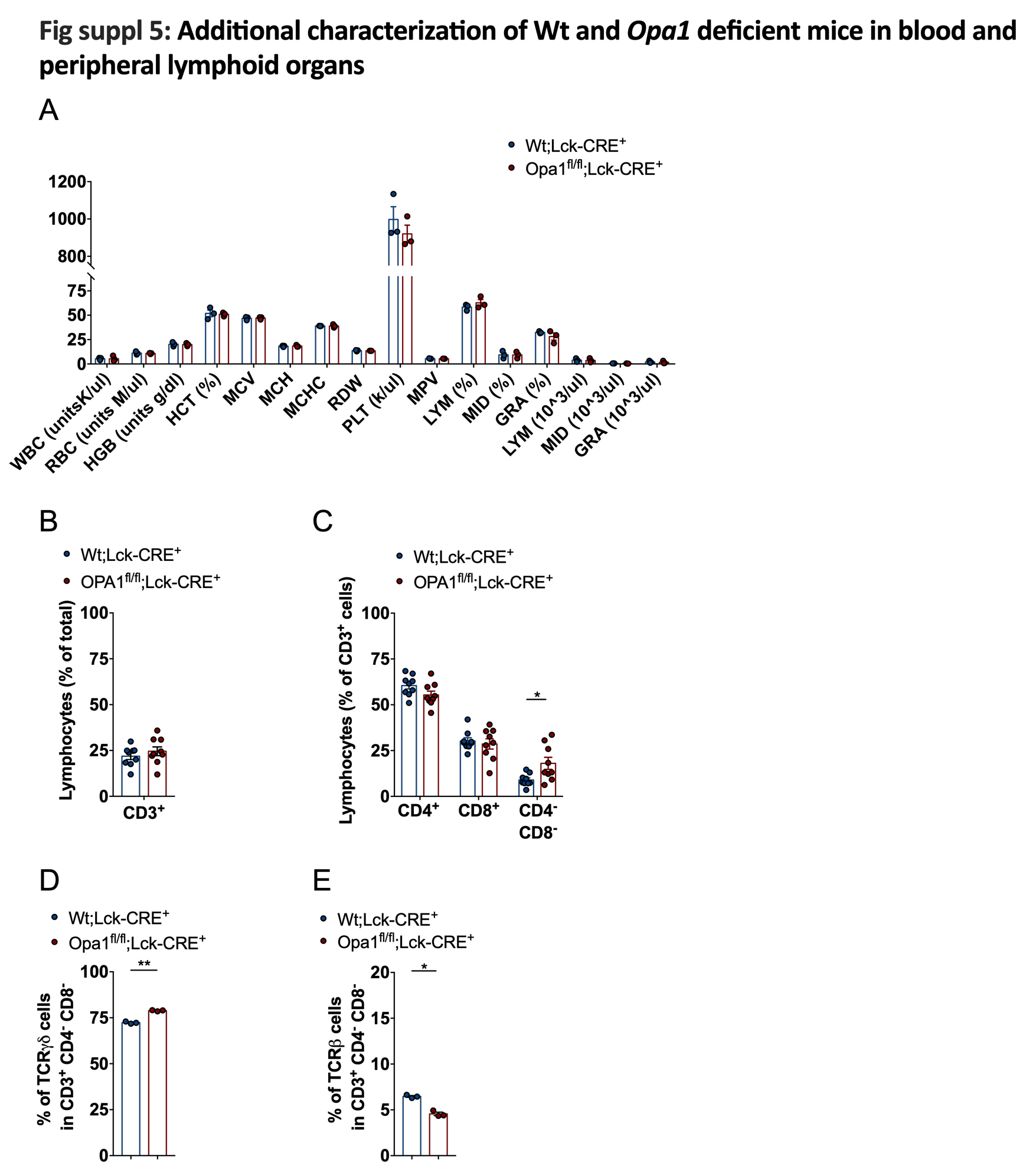


**Supplementary Figure 9: Additional characterization of Wt and *Opa1* deficient mice in blood and peripheral lymphoid organs.**

1. Complete blood counts of mice of the indicated genotype. Data are mean ± SEM (N=3/group)
2. Frequencies of CD3+ splenocytes in mice of the indicated genotype. Each dot represents an individual mouse. Data are mean ± SEM (N=9/group)
3. Frequencies of CD4+, CD8+ and CD4- CD8- splenocytes in in mice of the indicated genotype. Each dot represents an individual mouse. Data are mean ± SEM (N=9/group). *, P<0.05
4. Frequencies of TCRγδ+ subsets in CD3+ CD4- CD8- lymphocytes isolated from mice of the indicated genotype. Data are mean ± SEM (N=3/group). **, P<0.01
5. Frequencies of TCRβ+ subsets in CD3+ CD4- CD8- lymphocytes isolated from mice of the indicated genotype. Data are mean ± SEM (N=3/group). *, P<0.05
